# Network analysis of master regulators associated with invasive phenotypes in multiple myeloma

**DOI:** 10.3389/fcell.2025.1586870

**Published:** 2025-07-16

**Authors:** Feng Qian, Yubo Wang, Qinghong Shi

**Affiliations:** ^1^ Department of Anesthesiology, China-Japan Union Hospital of Jilin University, Changchun, China; ^2^ Department of Clinical Laboratory, China-Japan Union Hospital of Jilin University, Changchun, China

**Keywords:** EMM, invasiveness, master regulators, erg, idarubicin

## Abstract

To elucidate the role of transcriptional regulators (TRs) associated with invasiveness in multiple myeloma (MM), we conducted a systematic network analysis to identify key master regulators (MRs) that govern MM invasiveness. We employed a consensus clustering method based on a 24-gene signature to classify MM patients into high invasiveness (INV-H) and low invasiveness (INV-L) groups. Subsequently, we identified TRs specific to the INV-H and INV-L phenotypes as MRs using a network-based approach, and we validated the MR activities that correlated with the INV-H phenotype across multiple independent datasets. We evaluated the effect of MRs on patient outcomes in relation to the prognosis of MM. By utilizing siRNA to disrupt ERG expression in U266 and RPMI8226 cell lines, we evaluated the effects of the master regulator ERG on the proliferation, apoptosis, invasion, and migration of myeloma cell lines, and we confirmed the expression of ERG in patients with extramedullary MM. We assessed invasiveness using a 24-gene signature, categorizing patients into INV-H and INV-L groups. Our network identified MRs linked to MM invasiveness and revealed enriched signaling pathways. High ERG expression correlated with poor prognosis. ERG silencing reduced cell invasiveness, migration, and apoptosis, while promoting proliferation. Elevated ERG was found in extramedullary MM, and potential drug candidates, including Idarubicin, were identified for treatment. This study provides a comprehensive analysis of master regulators in EMM, contributing to targeted therapeutic strategies. We identified ERG as a marker for extramedullary invasion in MM, suggesting it as a potential therapeutic target for future interventions.

## Introduction

Extramedullary multiple myeloma (EMM) is a hematologic malignancy characterized by the abnormal proliferation of plasma cells in tissues or organs outside the bone marrow (Bansal et al., 2021). Compared to bone marrow-based multiple myeloma (MM), the treatment of EMM is more challenging, and the prognosis is generally poor (Bladé et al., 2022). The mechanisms underlying the development of EMM are not yet fully understood (Zanwar et al., 2023), but studies suggest that its progression is closely related to gene mutations and chromosomal abnormalities, such as 17p deletion and t (4; 14) translocation. Further, disease progression in EMM patients is often accompanied by complications such as hypercalcemia, renal dysfunction, and anemia, which severely impact the patients’ quality of life and survival (Bhutani et al., 2020). The diagnosis of EMM relies on imaging techniques (e.g., CT, MRI), hematological tests, and bone marrow biopsy. Imaging helps identify the distribution of tumors in soft tissues or extra-bone organs, while bone marrow biopsy allows for direct observation of tumor cells in the bone marrow. Treatment typically involves a combination of chemotherapy, targeted therapy, and immunotherapy (Bladé et al., 2011).

The pathogenesis of EMM remains incompletely understood; however, it is closely associated with high-risk genetic abnormalities and alterations in the tumor microenvironment (Gong et al., 2024). The prognosis for patients with EMM is poor, conventional treatments exhibit limited efficacy (Strauss et al., 2023). The median overall survival (OS) for patients with primary extramedullary invasion is 43.6 months, whereas the median OS for those with secondary extramedullary invasion is only 8.4 months (Zhang et al., 2022). Further, patients presenting with more than one extramedullary lesion demonstrate a significantly worse prognosis, with 3-year survival rates of 28.1% and 59.2%, respectively (Zanwar et al., 2023). The type and number of extramedullary lesions have a substantial impact on prognosis, as studies indicate that patients with multiple extramedullary lesions experience a markedly poorer outcome.

In this study, we employed a consensus clustering method based on a 24-gene signature to categorize MM patients into INV-H and INV-L groups. Utilizing network methods, we identified transcriptional regulators (TRs) specific to these two phenotypes and validated their association with the INV-H phenotype across multiple independent datasets. Further prognostic analysis indicated that high expression of ERG was closely correlated with poor prognosis in MM. We assessed the function of ERG in U266 and RPMI8226 myeloma cell lines using siRNA interference and discovered that the inhibition of ERG significantly reduced the invasion and migration capabilities of the cells. Additionally, ERG was found to promote the proliferation of MM cells while inhibiting apoptosis. Additionally, we identified potential new drug candidates, including Idarubicin, for the treatment of EMM. Notably, we observed high expression of ERG in extramedullary MM samples, suggesting that ERG may play a critical role in the invasiveness and prognosis of MM.

## Materials and methods

### Data collection and preprocessing

This study utilized two datasets from the GEO database: GSE39754 and 2GSE7213. GSE39754 contains gene expression profiles from 170 newly diagnosed MM patients. Although explicit annotation for EMM or invasive subtypes is not provided, we stratified patients into high- and low-invasiveness groups based on the expression profiles of curated invasion-associated genes, to serve as a surrogate marker for invasive potential. To ensure comparability of gene expression levels across different samples, we performed log2 normalization for subsequent analysis. The normalization process ensured that gene expression levels between different samples could be fairly compared. Additionally, we plotted box plots to verify that the normalized data had good distribution characteristics. For the GSE72213 dataset, which contains 29 samples including both MM patients and healthy controls, it was primarily used for weighted gene co-expression network analysis (WGCNA) to identify gene modules significantly associated with MM phenotype status. To ensure consistency and accuracy in data analysis, we used the normalized data for all subsequent analyses.

### Cancer invasiveness clustering

To investigate the heterogeneity of invasiveness phenotypes among MM patients, we utilized a previously published 24-gene invasiveness-related signature derived from a comprehensive pan-cancer multi-omics analysis (Bi et al., 2021). This signature includes COL11A1, POSTN, EPYC, ASPN, COL10A1, THBS2, FAP, LOX, SFRP4, INHBA, MFAP5, GREM1, COMP, VCAN, COL5A2, COL5A1, TIMP3, GAS1, TNFAIP6, ADAM12, FBN1, SULF1, COL1A1, and DCN, which represent core molecular determinants of cancer invasiveness. An expression matrix of these genes was constructed from the GSE39754 dataset, and consensus clustering was applied to stratify MM patients into distinct invasiveness subtypes based on their expression patterns. These genes were subjected to RNA sequencing data screening, normalization, and log2 transformation to ensure normal distribution and comparability of the data. Next, we performed unsupervised consensus clustering using the ConsensusClusterPlus R package, calculating the distances between samples using Euclidean distance and clustering the samples using the hierarchical clustering algorithm (ward. D2). To ensure the stability and reliability of the clustering results, we set the maximum number of clusters to 6 and performed 5000 resampling. Finally, the samples were divided into three subgroups: INV-H, INV-M, and INV-L, representing different invasiveness phenotypes. While three subtypes were identified, we focused on the comparison between INV-H and INV-L to highlight the most divergent transcriptional patterns related to invasion.

### Inferring gene regulatory networks

To understand the regulatory mechanisms behind gene expression patterns, identify potential master regulators (MRs), and explore their roles in disease progression, constructing and analyzing gene regulatory networks (GRNs) is a crucial step. We employed two advanced machine learning techniques RGBM and ARACNE to infer high-quality GRNs. RGBM is a feature selection-based method that can capture both linear and nonlinear interactions between transcriptional regulators (TRs) and target genes, while ARACNE (Lachmann et al., 2016) is based on mutual information theory, preventing indirect transmission of interactions and providing statistical significance for TR-target gene interactions. These two methods were implemented using the RGBM (Mall et al., 2018) (v1.0.10) and corto (v1.1.11) packages in R, and transcriptional regulators with a target gene size smaller than 10 were removed from the inferred GRNs. Quality control steps ensured that the inferred GRNs were highly accurate and reliable. These GRNs, constructed based on the GSE39754 dataset, provided a robust framework for downstream master regulator analysis.

### Scoring TR activities

For each sample, the activity of the transcriptional regulator (TR) is estimated based on the collective mRNA levels of its target genes (Paull et al., 2021). To estimate the activity of TRs in each sample, we combined two methods: RGBM and VIPER. The RGBM method calculates the Pearson correlation coefficient between TRs and their target genes, classifying the target genes into activation targets and repression targets, and computes the TR activity score using the following formula:

$$\text{Ac}t{\left(TR,i\right)}^{C}=\frac{1}{u}\sum_{k=1}^{u}t_{ki}^{p}-\frac{1}{v}\sum_{j=1}^{v}t_{ji}^{n}$$

The VIPER method uses a probabilistic framework to directly integrate the regulatory patterns of target genes and the confidence of interactions, calculating the enrichment score (NES) of TR regulons. The higher the NES score, the more enriched the TR regulon is in the sample, indicating higher TR activity [[6,7]]. The effective combination of both methods provides strong support for identifying differentially activated TRs (MRs). To further validate the TR activity scores, we applied the same scoring method in the independent dataset GSE72213 and experimentally validated the activity patterns of key TRs.

### Gene set enrichment analysis and MR selection

To identify key master regulators (MRs) specific to INV-H and INV-L, we used three different gene set enrichment/activity estimation techniques: FGSEA, GSVA, and VIPER, in combination with two GRN inference techniques: RGBM and ARACNE. To ensure robustness, four MRA strategies were applied: (1) RGBM + FGSEA, (2) RGBM + GSVA, (3) ARACNE + VIPER, and (4) ARACNE + GSVA. Only master regulators (MRs) identified by all four methods were retained as consensus MRs for downstream analysis of INV-H and INV-L phenotypes (Califano and Alvarez, 2017; Lim et al., 2009). To further explore the functions and biological significance of these MRs, we performed downstream pathway enrichment analysis on 126 INV-H-specific MRs using the ConsensusPathDB framework. Similarly, we conducted enrichment analysis on the 10 INV-L-specific MRs.

### Immune cell infiltration assessment

To investigate the potential role of the ERG gene in the tumor microenvironment, we employed the CIBERSORT algorithm to infer the immune cell composition in samples from the GSE72213 dataset. CIBERSORT is a deconvolution method based on RNA-seq data that accurately estimates the relative abundance of 22 immune cell types within a sample. All data were normalized to ensure comparability across different samples. Furthermore, to assess the correlation between ERG gene expression levels and the extent of immune cell infiltration, we calculated the Spearman rank correlation coefficient (Spearman R) between ERG expression values and the infiltration proportions of various immune cell types. Spearman rank correlation analysis was conducted to determine the relationships between ERG gene expression and various immune cells, with significant correlations defined as those with P < 0.05.

### Culture of U266 and RPMI8226 cell lines

Both U266 and RPMI8226 cell lines were cultured in RPMI-1640 medium supplemented with 10% fetal bovine serum and 1% penicillin-streptomycin solution. Cells were cultured at 37°C with 5% CO_2_ in a constant temperature incubator, with regular observation of cell growth. When cell density reached 80%–90%, cells were passaged using 0.25% trypsin, and the cell suspension was transferred to a new culture flask for further culturing.

### siRNA-ERG interference in U266 and RPMI8226 cells

Based on the ERG gene sequence, specific siRNAs targeting the gene were selected, designed, and synthesized. The sequences of the siRNA were:

F: CGGAGUCAUCUCUGUACAATT.

R: UUGUACAGAGAUGACUCCGTT.

U266 or RPMI8226 cells were seeded in 6-well plates and cultured to 80% confluence. Transfection was performed with Lipofectamine 2000, using siRNA mixed with the reagent in serum-free medium. After gentle mixing, the mixture was added to the cells and cultured for 48 h. Target gene expression was assessed by Western blot to confirm siRNA interference.

### Transwell invasion and migration assay

The invasion and migration abilities of U266 and RPMI-8226 cells were evaluated using the Transwell assay. For invasion, 1 × 10^5^ RPMI-8226 cells were seeded in the upper chamber coated with Matrigel and cultured in serum-free medium, while the lower chamber contained medium with 10% FBS. After 24 h, cells were counted under a microscope, and the number of invaded cells was calculated. For migration, cells from the logarithmic growth phase of both the control and siRNA-ERG knockdown groups were resuspended in serum-free medium, with 1 × 10^5^ cells added to the upper chamber. The lower chamber contained medium with 10% FBS, and after 24 h, cells in the lower chamber were counted.

### CCK8 proliferation assay

U266-ctrl, U266-siRNA-ERG, RPMI8226-ctrl, and RPMI8226-siRNA-ERG cells were seeded in 96-well plates at 5000 cells per well and cultured at 37°C, 5% CO_2_ until logarithmic growth. 10 μL of CCK-8 reagent was added to each well, and the plates were incubated for 4 h. The WST-8 reaction produced an orange-yellow formazan product, with color intensity reflecting cell viability. Absorbance (OD) was measured at 450 nm using a microplate reader. OD values correlated with cell viability or proliferation, and comparisons were made to assess changes in proliferation.

### Annexin V/7AAD apoptosis assay

U266-ctrl, U266-siRNA-ERG, RPMI8226-ctrl, and RPMI8226-siRNA-ERG cells were seeded in 6-well plates at 5 × 10^5^ cells per well. 1 × 10^6^ cells were transferred to a new tube, and Annexin V-FITC and 7AAD staining solutions were added (5 μL of each, along with Binding Buffer). The cells were incubated in the dark for 10 min at room temperature, then diluted with PBS. Fluorescence intensity was analyzed by flow cytometry.

### IHC detection of ERG expression in EMM tumor tissues

EMM tumor tissue samples fixed in 4% formaldehyde for 24 h, and embedded in paraffin. Perform IHC staining procedure use anti-ERG primary antibody, Then secondary antibody for 30 min. DAB staining revealed ERG expression. Slides were analyzed microscopically based on staining intensity and positive cell percentage.

### ERG drug docking

In an effort to improve the prognosis and overall survival rate of the disease, we attempted to investigate the interaction between 3015 drugs of Food and Drug Administration (FDA) database and ERG. The molecular docking program AutoDock Tools (Morris et al., 2009) and AutoDock vina (Trott and Olson, 2010) was used for the automated molecular docking simulations. The 3D structure of ERG (P11308) protein was obtained from Uniprot database (https://www.uniprot.org/). We have used Discovery Studio Visualizer to visualize and analyze the interactions. Prior to molecular docking calculations, water molecules and the ligand in the protein structure were removed, and hydrogens and Gasteiger charges were added. Ten docking poses were obtained for molecular docking calculation.

## Result

### Identification of invasive features in extramedullary MM

**FIGURE 1:**
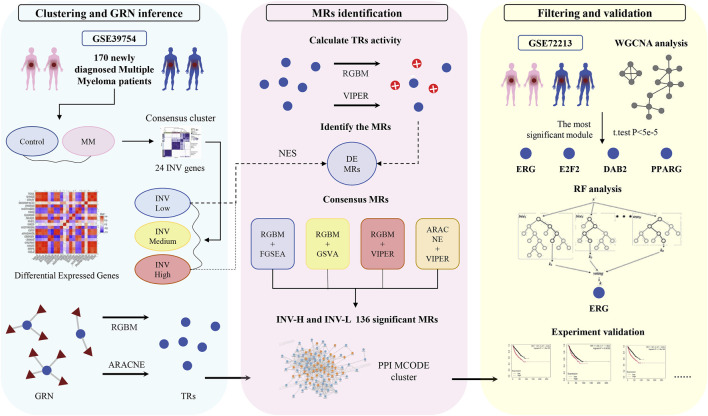
Flowchart of this study. In clustering and GRN inference, the samples were labeled as INV-H, INV-M, and INV-L based on the features of 24 invasion-related genes.

**FIGURE 2:**
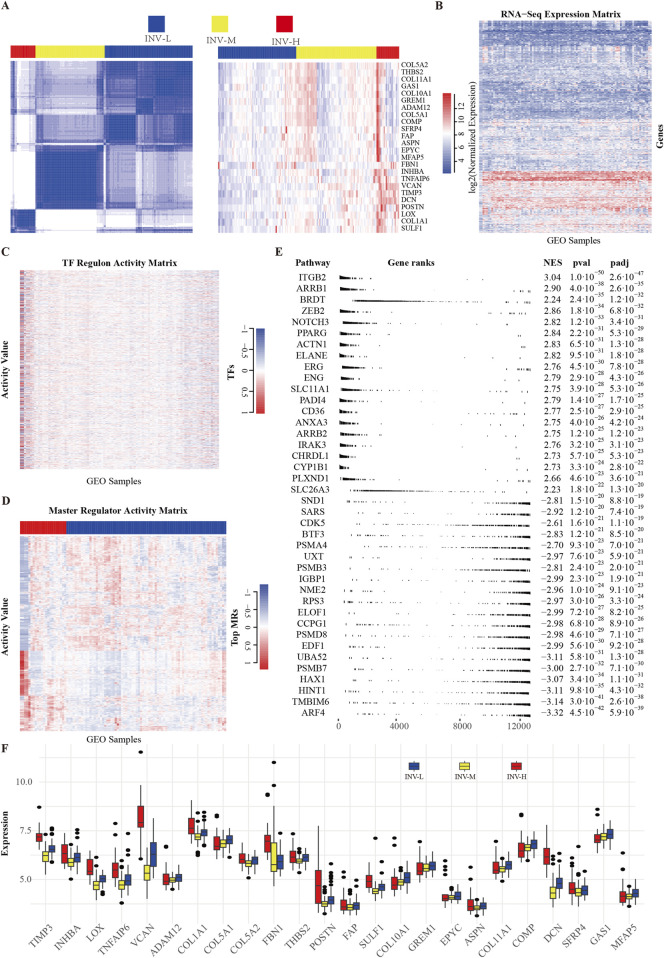
Screening and Clustering of Invasion Gene Set. **(A)** Unsupervised consensus clustering results and heatmap of 24 invasion-related genes. **(B)** Heatmap of gene expression matrix from the GEO dataset. **(C)** Transcription factor regulatory activity matrix. The transcription factors here include all those with a regulon size greater than or equal to 10. **(D)** Activity matrix of key regulatory factors (MRs), with the top 5% highly variable genes filtered based on expression variability (IQR). **(E)** Enrichment results of top MRs. Multi-level enrichment analysis was conducted using the fgseaMultilevel function, selecting the top 20 gene sets with the highest and lowest enrichment scores. **(F)** Expression values of 24 invasion-related genes in high, medium, and low groups of samples.

To explore gene expression differences among various invasive phenotypes, we analyzed the expression profiles of a previously reported 24-gene invasiveness signature within the GSE39754 dataset. Figure 1 provides an overview of the entire processing pipeline. We constructed an expression heatmap (Figure 2A) to illustrate the expression variations of these genes across different invasive samples. To validate the robustness and reliability of the clustering results and to accurately differentiate among the invasive phenotypes, we employed an unsupervised consensus clustering method, which ultimately categorized the samples into three groups: “high invasiveness” (INV-H), “moderate invasiveness” (INV-M), and “low invasiveness” (INV-L) (Figure 2A; Supplementary Figure 1). We also presented the RNA-Seq expression matrix (Figure 2B) and the transcription factor activity matrix (Figure 2C). The master regulatory factors (MRs) extracted from the activity matrix exhibited a distinct block diagonal structure (Figure 2D), indicating that certain MRs are more active in INV-H samples, whereas their activity is diminished in INV-L samples. This finding suggests significant differences in the activity patterns of MRs across the various invasive phenotypes.

We performed multilayer enrichment analysis using FGSEA to examine gene set enrichment across invasive phenotypes. The top 20 gene sets with the highest and lowest enrichment scores were selected (Figure 2E). Additionally, we analyzed the distribution of 24 invasion-related genes across high, low, and moderate INV groups, providing a detailed gene expression profile for each phenotype (Figure 2F).

### Activity analysis of MRs

**FIGURE 3:**
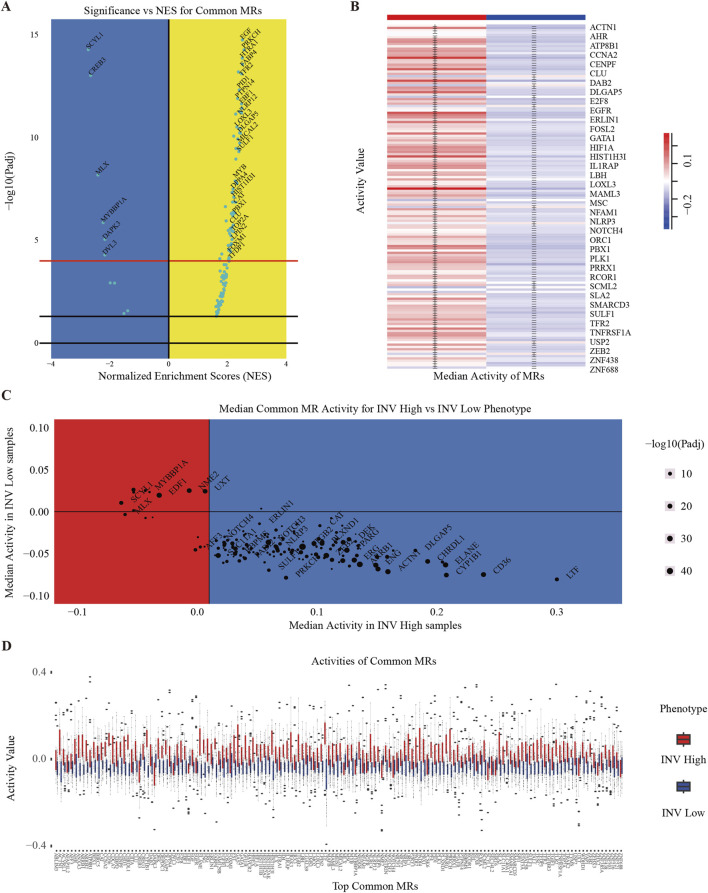
Clustering of INV Gene Set. **(A)** Volcano plot of the most significantly different transcription factors (TFs). Shows statistical significance and effect size for each gene in the gene expression data. The x-axis represents the log-transformed *P*-values, and the y-axis represents the NES score. **(B)** Heatmap to show the median activity of multiple MRs in MM. **(C)** Scatter plot showing the average activity of different MRs in two phenotypes (INV High and INV Low) and distinguishing significance by color and size. **(D)** Activity values of multiple MRs in MM, grouped by phenotype (INV High and INV Low).

To quantify and visualize the activity differences among these MRs, we employed a volcano plot to illustrate the normalized enrichment scores (NES) of the common MRs. The x-axis represents the NES of transcriptional regulators, while the y-axis indicates the enrichment significance in the context of MM cancer (Figure 3A). Transcriptional regulators positioned above the “red” line are classified as MRs, signifying enhanced activity in specific samples. Furthermore, to gain deeper insights into the distribution of these MRs across various invasive phenotypes, we presented the classification results of these MRs in tumor samples exhibiting different levels of invasiveness (Supplementary Figure 2). We also illustrated the activity distribution of the top MRs in samples categorized as INV-H and INV-L (Figure 3D). Further, we validated the activity of master regulators specific to INV-H and INV-L in MM (Supplementary Figure 3). The findings reveal that these MRs display significantly distinct activity patterns across various invasive phenotypes, suggesting their potential roles at different stages of tumor progression.

**FIGURE 4:**
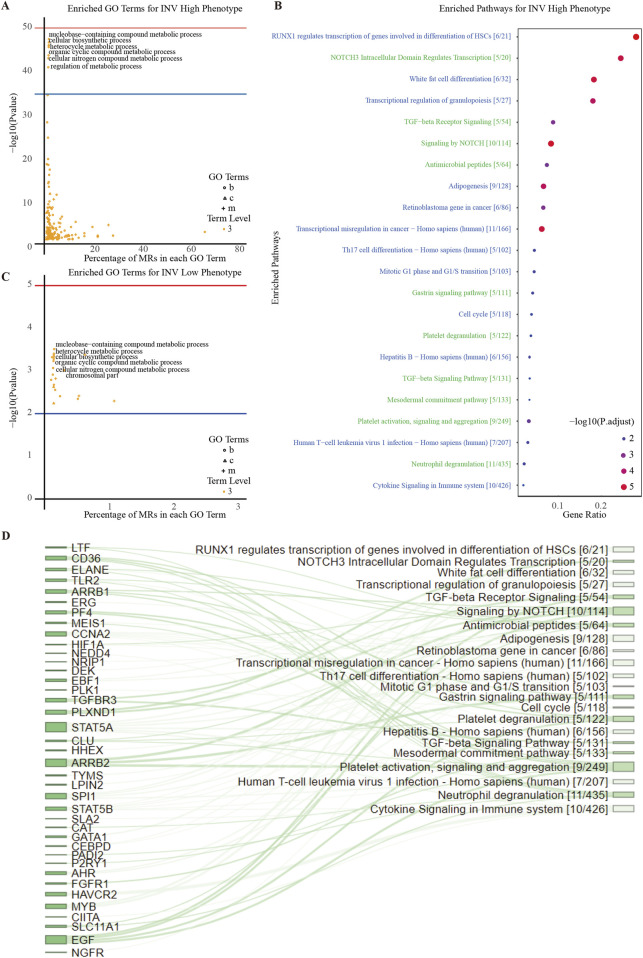
Enrichment Analysis of INV Gene Set. **(A)** Downstream GO term enrichment and pathway enrichment for INV-high-specific MRs. **(B)** Downstream GO term enrichment and pathway enrichment for INV-low-specific MRs. **(C)** Pathways enriched in the INV High phenotype and their associated statistical information. **(D)** Sankey diagram showing the relationships between INV-high-specific MRs and pathways. The larger the node area, the more enriched the connected MRs or pathways.

To illustrate MR expression across invasive phenotypes, we plotted MR expression in INV-H and INV-L samples (Figure 3B) and created a volcano plot to identify significantly differentially expressed MRs (Figure 3C). This analysis revealed distinct MR activity and expression patterns across phenotypes, suggesting potential biomarkers or therapeutic targets. We identified 136 significant MRs, with 126 showing higher activity in INV-H samples. GO and pathway enrichment analysis revealed that INV-H MRs were enriched in inflammation and immune response pathways (Figures 4A,B), while INV-L pathways involved melanogenesis and insulin resistance (Figure 4C). These results highlight potential therapeutic targets for highly invasive cancers.

### Validation of MRs activity patterns

**FIGURE 5:**
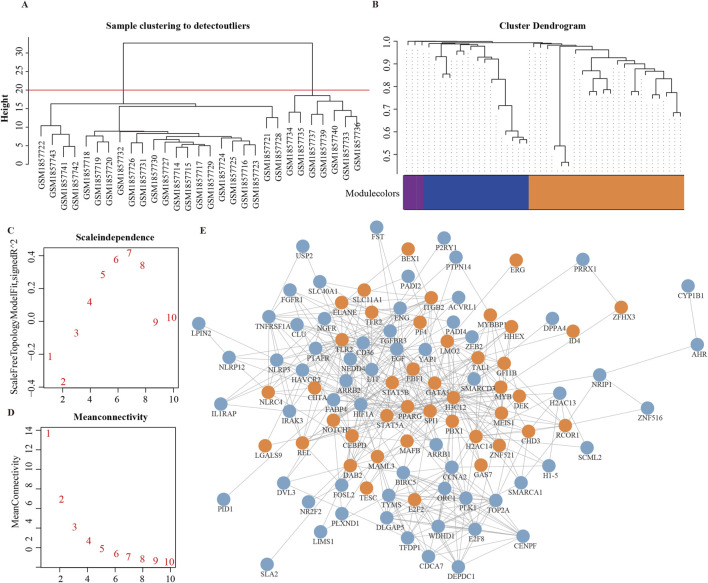
Validation of INV Gene Set. **(A)** Hierarchical clustering of GSE72213 dataset samples for outlier detection. Euclidean distance and average linkage were used to construct the sample dendrogram. A height threshold of 20 (red line) was applied, and seven outlier samples falling outside the main cluster were removed before WGCNA. **(B)** Final module color map after soft-threshold selection and module identification. **(C, D)** Soft-threshold selection for module identification. **(E)** Protein-protein interaction network of 136 significant MRs. Orange nodes are the highest-scoring MRs based on MOCDE clustering, and blue nodes are MRs outside the clustering.

To comprehensively validate the activity patterns of MRs, we utilized an independent dataset, GSE72213. Initially, we constructed a protein interaction network for the 136 significant MRs obtained from GSE39754. Using the MCODE clustering algorithm, we extracted the most densely connected subnetwork, consisting of 42 genes (Figure 5E). We subsequently applied WGCNA to identify gene modules significantly associated with the MM phenotype. After excluding outlier samples, we selected the module with the highest module-phenotype correlation (ME2, r = 0.815), which revealed 15 genes significantly related to the MM phenotype. Among these, we focused on genes with a p-value less than 5e-5, determined by a two-sample t-test, to ensure high significance (P < 5e-5 is commonly employed in large-scale genomic studies to control false positive rates and enhance the robustness of results). Ultimately, we identified four key genes: ERG, E2F2, DAB2, and PPARG (Figures 5A–D). A random forest analysis was conducted to assess the predictive importance of these genes, and following 10-fold cross-validation, ERG emerged as the most significant gene (Supplementary Figure 4), highlighting its potential as a core gene with clinical relevance. To further validate feature importance, we applied SHAP analysis, which confirmed ERG as the top contributor with the highest average SHAP value (0.024), thereby reinforcing its robustness as a predictive biomarker.

### The impact of ERG on EMM prognosis

**FIGURE 6:**
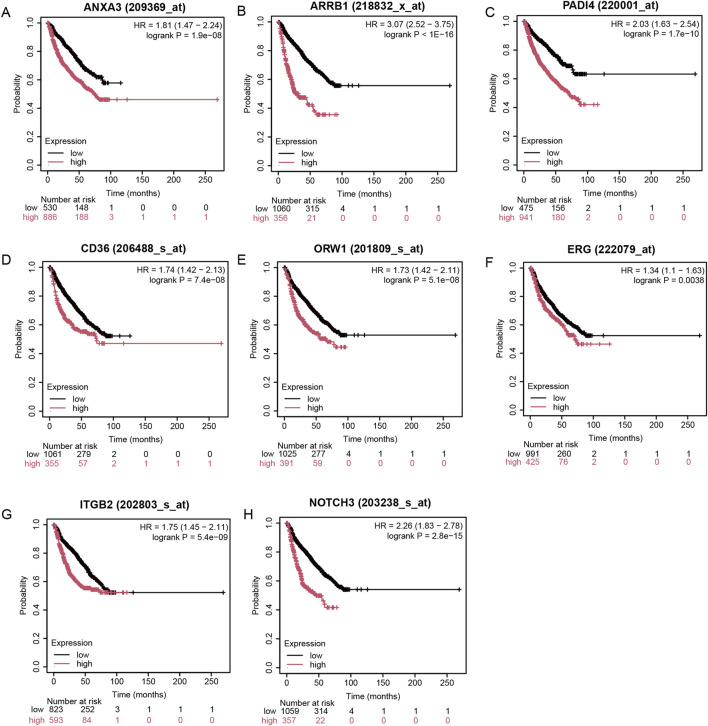
Impact of ERG on MM prognosis. **(A-H)** High expression of eight regulatory factors indicates poor prognosis in MM.

To further validate the prognostic value of ERG, we utilized the Kaplan-Meier Plotter database to examine the relationship between ERG expression and the survival of MM patients. Our results indicated that patients exhibiting high ERG expression experienced significantly shorter survival times (Figures 6A–H). This finding not only corroborates the prognostic significance of ERG in MM but also suggests that ERG may serve as a potential therapeutic target, aligning with the outcomes of our prior analyses. Furthermore, we investigated the prognostic implications of 23 additional MRs and discovered that elevated expression levels of genes such as ANXA3, ARRB1, PADI4, CD36, ORW1, ITGB1, and NOTCH3 were also linked to poor prognosis (Figures 6A–H).

### Correlation analysis of ERG gene and immune cell infiltration

**FIGURE 7:**
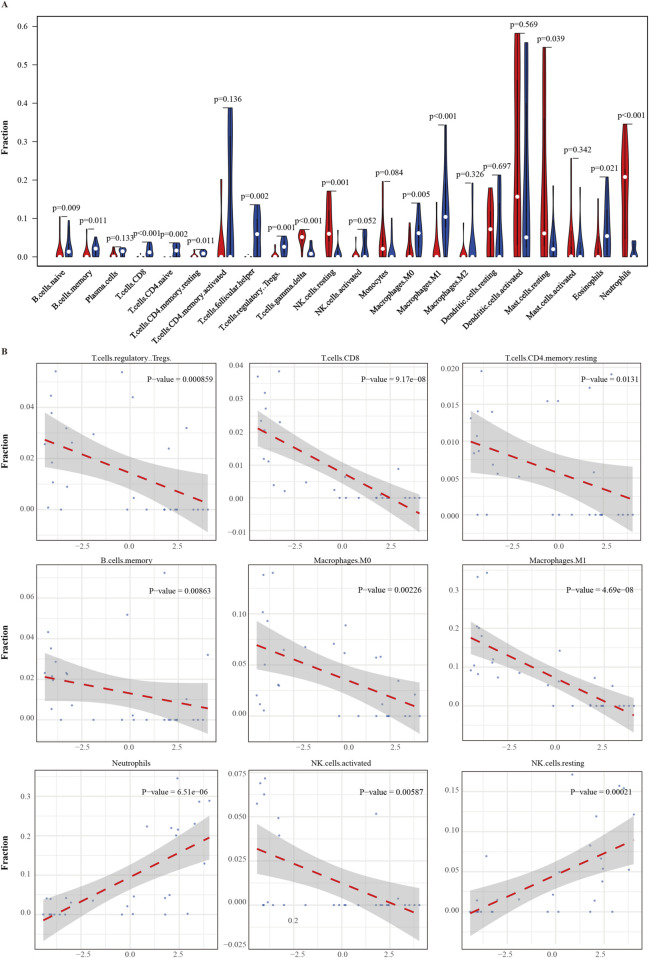
Correlation between ERG gene and Immune cell infiltration. **(A)** correlation between ERG expression and infiltration of 22 immune cell types in the tumor microenvironment. **(B)** Curve of the correlation between ERG and immune cells.

To explore the potential immune regulatory role of the ERG gene in the tumor microenvironment, we analyzed the correlation between ERG expression levels and the infiltration levels of 22 immune cell types (Figure 7A). The results demonstrated significant correlations between ERG expression and the infiltration levels of various immune cells (P < 0.05), including memory B cells, naïve B cells, CD8^+^ T cells, naïve CD4^+^ T cells, resting memory CD4^+^ T cells, and follicular helper T cells, among others. Specifically, ERG expression levels were negatively correlated with CD8^+^ T cells (*R* = −0.812, *P* = 9.17 × 10^−8^), M1 macrophages (*R* = −0.822, *P* = 4.69 × 10^−8^), follicular helper T cells (*R* = −0.708, *P* = 1.73 × 10^−5^), naïve CD4^+^ T cells (*R* = −0.709, *P* = 1.69 × 10^−5^), and Treg cells (*R* = −0.585, *P* = 8.59 × 10^−4^), suggesting that high ERG expression may inhibit the infiltration of these immune cells. Additionally, ERG expression was also negatively correlated with M0 macrophages (*R* = −0.545, P = 0.002), NK cells (*R* = −0.499, *P* = 0.006), memory B cells (*R* = −0.479, *P* = 0.009), and naïve B cells (*R* = −0.501, *P* = 0.006), indicating that ERG may play a crucial role in regulating the polarization and differentiation of immune cells. Conversely, ERG expression levels were significantly positively correlated with certain immune cells, including γδT cells (*R* = 0.733, *P* = 6.16 × 10^−6^), neutrophils (*R* = 0.732, *P* = 6.51 × 10^−6^), resting NK cells (*R* = 0.636, *P* = 2.10 × 10^−4^), and activated mast cells (*R* = 0.268, *P* = 0.160). These results suggest that ERG may play a role in promoting the recruitment and activation of specific innate immune cells (Figure 7B; Supplementary Figure 5).

### The impact of ERG on MM cell function

**FIGURE 8:**
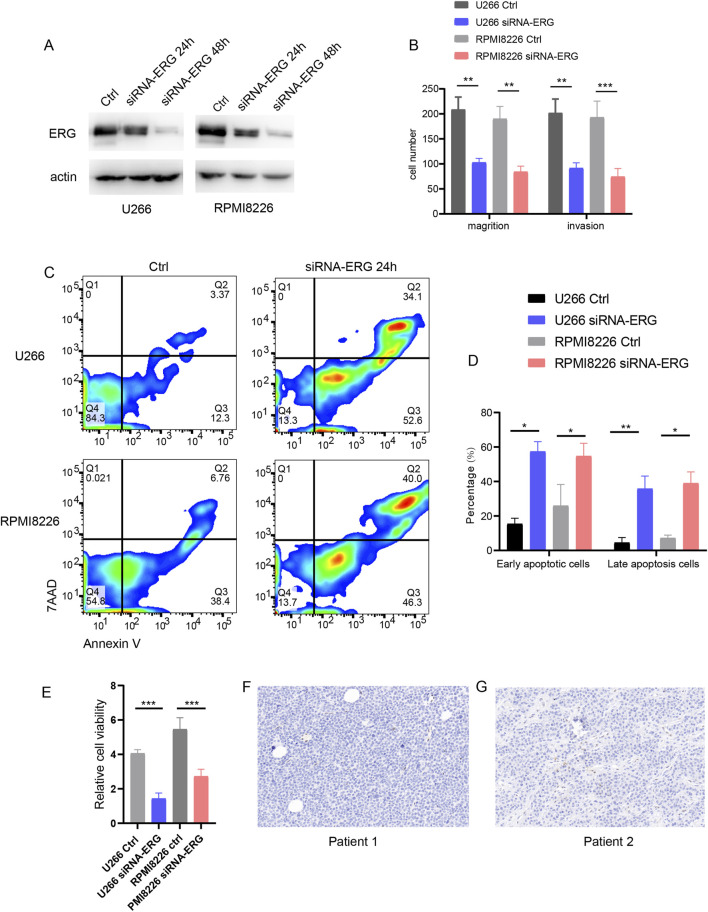
ERG effect on MM function. **(A)** WB detection of interference efficiency after ERG was knocked down with siRNA in U266 and RPMI8226 cells. **(B)** Effect of ERG expression on invasion and migration of U266 and RPMI8226 cells. **(C)** Annexin V/7AAD detection of cell apoptosis. **(D)** Statistical chart of early and late apoptosis. **(E)** CCK8 assay for cell proliferation. **(F, G)** Expression of ERG in EMM patient tissues.

Literature research has demonstrated that the expression and function of the ERG gene hold significant clinical implications in MM. The ERG gene is a member of the ETS transcription factor family and plays a crucial role in hematopoiesis and angiogenesis. Previous studies have indicated that ERG promotes tumor initiation and progression in prostate cancer, lung cancer, and acute myeloid leukemia (AML); however, its specific effects in MM remain inadequately understood. To investigate this, we employed siRNA to interfere with ERG expression in the U266 and RPMI8226 cell lines, validating the interference efficiency through Western blotting (Figure 8A). The experimental results indicated that reducing ERG protein expression diminished the invasion and migration capabilities of the myeloma cell lines (Figure 8B). Furthermore, using Annexin V/PI staining to evaluate the impact of ERG on MM, we observed that low ERG expression enhanced both early and late apoptosis in U266 and RPMI8226 cells (Figures 8C,D). CCK8 assays further revealed that decreased ERG expression inhibited the proliferation of these cell lines (Figure 8E). Additionally, we identified a population of ERG-high-expressing cells in EMM tissue samples (Figure 8F), underscoring the significant role of ERG in extramedullary invasion and the development of MM.

### Drug screening based on ERG as a target

**FIGURE 9:**
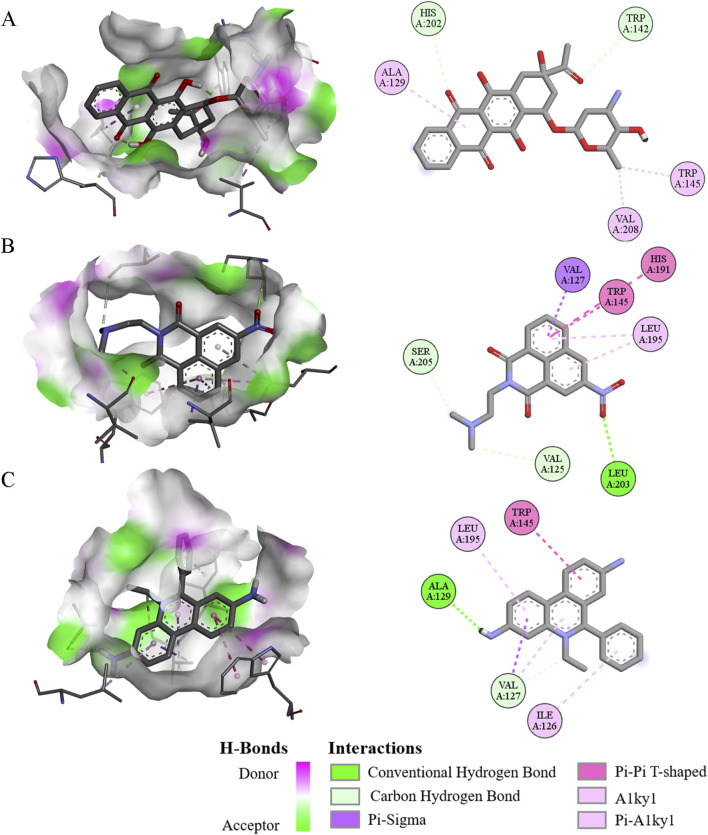
The 3D and 2D interaction diagram of ERG protein with three compounds. **(A–C)** Visualization of molecular docking of ERG protein with Idarubicin **(A)**, Mitonafide **(B)**, and Homidium bromide **(C)** binding pockets, respectively.

From a functional perspective, the ERG protein may serve as an excellent target for screening novel drug candidates for MM. To investigate this, the receptor-ligand interactions between the ERG protein and each of the 3,015 compounds were analyzed using molecular docking. This approach led to the identification of the top five drugs that interact with the amino acid residues of the ERG protein and exhibit the lowest docked binding affinities (Supplementary Table 1). Interestingly, the interactions between the top three compounds(Idarubicin, Mitonafide, and Homidium bromide) and the ERG protein demonstrated relatively higher binding affinities. Specific residues were found to form hydrogen bonds with these three compounds: Trp142 and His202 with Idarubicin (Figure 9A), Val125, Leu203, and Ser205 with Mitonafide (Figure 9B), and Val127 and Ala129 with Homidium bromide (Figure 9C). From a physicochemical properties’ standpoint, idarubicin exhibited a superior performance profile compared to Mitonafide and Homidium bromide (Supplementary Table 2). Therefore, Idarubicin (C_26_H_27_NO_9_) appears to be the most relevant structure among the small molecule drugs screened and represents a promising candidate for the treatment of MM.

## Discussion

EMM is an aggressive disease with a poor prognosis, facing challenges such as drug resistance and the absence of a standardized treatment regimen. Current treatment strategies typically include intensified induction combination therapy, autologous hematopoietic stem cell transplantation (ASCT), and dual-drug maintenance therapy (Tian et al., 2018). Novel drugs, including immunomodulatory agents, proteasome inhibitors, and immunotherapy, have demonstrated promising effects in enhancing prognosis, while molecular-targeted therapies and liquid biopsy techniques present new avenues for early diagnosis and treatment monitoring (Deng et al., 2021; Shi et al., 2024). Patients’ survival is influenced by various factors, including disease type, treatment response, age, and molecular genetic alterations. Genetic abnormalities, such as KRAS and NRAS mutations, long with chromosomal abnormalities, are closely associated with more aggressive disease and poor treatment responses (Shirazi et al., 2020). Therefore, understanding the mechanisms of invasion and the genetic characteristics of EMD is crucial for developing personalized treatment strategies.

Transcription factors play a crucial role in tumor invasion by regulating the expression of genes associated with cell proliferation, migration, invasion, angiogenesis, and immune evasion, thereby promoting malignant transformation and tumor progression (Li et al., 2019). These transcription factors modulate the activity of downstream signaling pathways including Wnt/β-catenin, Notch, HIF-1, and NF-κB, which facilitate the invasiveness and metastasis of tumor cells (Matthe et al., 2016). For example, transcription factors such as Twist, Slug, and Snail regulate epithelial-mesenchymal transition (EMT), enabling tumor cells to acquire enhance migration and invasion capabilities, thus promoting tumor metastasis. HIF-1 (hypoxia-inducible factor-1) plays a critical role in the tumor microenvironment under hypoxic conditions (Martin et al., 2011), assisting tumors in surviving and expanding by regulating angiogenesis factors like VEGF. Additionally, NF-κB enhances tumor invasiveness by promoting inflammatory responses and immune evasion. Aberrant expression of transcription factors is prevalent in various tumors; they not only act as key drivers of tumor initiation and progression but may also serve as potential therapeutic targets, contributing to the development of novel cancer treatment strategies (Jovanović et al., 2018).

Studies on the ERG gene in MM indicate that its expression is closely associated with the progression and prognosis of the disease. While Knief et al. reported ERG expression in MM as a diagnostic pitfall when distinguishing it from other ERG-positive tumors, such as prostate cancer, their study did not evaluate its prognostic significance (Knief et al., 2017). However, other findings support a functional role for ERG in MM pathogenesis. For example, miR-1179 has been shown to inhibit ERG expression and suppress the growth and proliferation of MM cells, implying that ERG overexpression may contribute to MM cell proliferation and invasion. Further, the expression of the ERG gene could serve as a potential diagnostic marker, as it is also highly expressed in other hematologic malignancies such as AML (Mullen et al., 2022). The aberrant expression of ERG is not limited to MM; it is also implicated in other hematologic malignancies, including acute lymphoblastic leukemia (ALL) and lymphoma (Yamashita et al., 2024).

In clinical practice, ERG gene testing is used to assess the prognosis of MM patients. In clinical practice, ERG gene testing is utilized to evaluate the prognosis of patients with MM. Research indicates that elevated expression of the ERG gene correlates with poor survival rates, suggesting that ERG may serve as a valuable prognostic biomarker (Culig, 2014). Targeted therapies aimed at the ERG gene are currently under development, with the goal of enhancing treatment outcomes for MM by inhibiting ERG gene activity. While the role of ERG in the pathogenesis of MM has been clarified, its expression patterns in other hematologic malignancies are intricate, necessitating careful differentiation of expression profiles in various disease contexts for practical application. Overall, investigations into the ERG gene in MM provide promising avenues for future diagnostic and therapeutic strategies (Donovan et al., 2019); however, additional studies are required to elucidate its role and clinical relevance.

The ERG protein presents a promising target for drug discovery in the treatment of MM. A recent study utilized molecular docking to examine the receptor-ligand interactions between the ERG protein and 3,015 different compounds, leading to the identification of several potential drug candidates (Pinzi and Rastelli, 2019; Crampon et al., 2022). Among the compounds tested, the top three—Idarubicin, Mitonafide, and Homidium bromide—showed strong interactions with key amino acid residues of the ERG protein. Idarubicin, in particular, demonstrated the highest binding affinity, with hydrogen bonds formed between the ERG residues Trp142 and His202 and the drug (Shahswar et al., 2024; Ohtake et al., 2011). Similarly, Mitonafide and Homidium bromide formed interactions with residues such as Val125, Leu203, and Ser205, and Val127 and Ala129, respectively. From a physicochemical perspective, Idarubicin outperformed the other compounds, showing a more favorable profile in terms of binding efficiency and interaction strength (Carella et al., 1990). Given its promising interaction with the ERG protein and its superior physicochemical properties (Cacciatore et al., 2024), Idarubicin stands out as a particularly promising candidate for further development as a treatment for MM. This research highlights the potential of targeted molecular docking as a powerful tool in identifying novel therapeutic agents for complex diseases like MM.

### Limitations

Several limitations warrant consideration. Our analyses utilized publicly available datasets with limited clinical annotation, resulting in an indirect assessment of invasiveness through gene expression clustering. Although integrative network algorithms were employed to identify master regulators, potential biases and parameter sensitivities may influence findings. The WGCNA was conducted on a modest sample size, which may affect module stability. Experimental validation of ERG was confined to *in vitro* models, and molecular docking predictions require further empirical substantiation. Lastly, validation in larger, well-annotated clinical cohorts is essential to confirm ERG’s prognostic and therapeutic relevance in multiple myeloma.

## Conclusion

In this study, we employed an integrative systems biology framework, combining transcriptional network analysis, master regulator inference, immune landscape profiling, and drug repurposing, to dissect the molecular determinants of invasive phenotypes in multiple myeloma. ERG emerged as a central regulator, associated with enhanced invasiveness, immune microenvironment remodeling, and potential therapeutic responsiveness. These findings, supported by both *in silico* and *in vitro* evidence, highlight ERG’s promise as a clinically relevant biomarker and therapeutic target in aggressive MM. Future efforts should aim to validate these biomarkers in larger patient cohorts and explore additional candidates using interpretable machine learning approaches. Such strategies may facilitate more precise risk stratification and pave the way for targeted interventions in high-risk myeloma.

## Data Availability

The original contributions presented in the study are included in the article/supplementary material, further inquiries can be directed to the corresponding author.

## Abbreviations

EMM, Invasiveness, Master regulators; ERG, Idarubicin.
