# Supplementary figures and images for: Network analysis of master regulators associated with invasive phenotypes in multiple myeloma
Source: Front Cell Dev Biol. 2025 Jul 16;13:1586870. doi: 10.3389/fcell.2025.1586870 (PMC12307296; doi:10.3389/fcell.2025.1586870)

# NES based classification of Common MRs

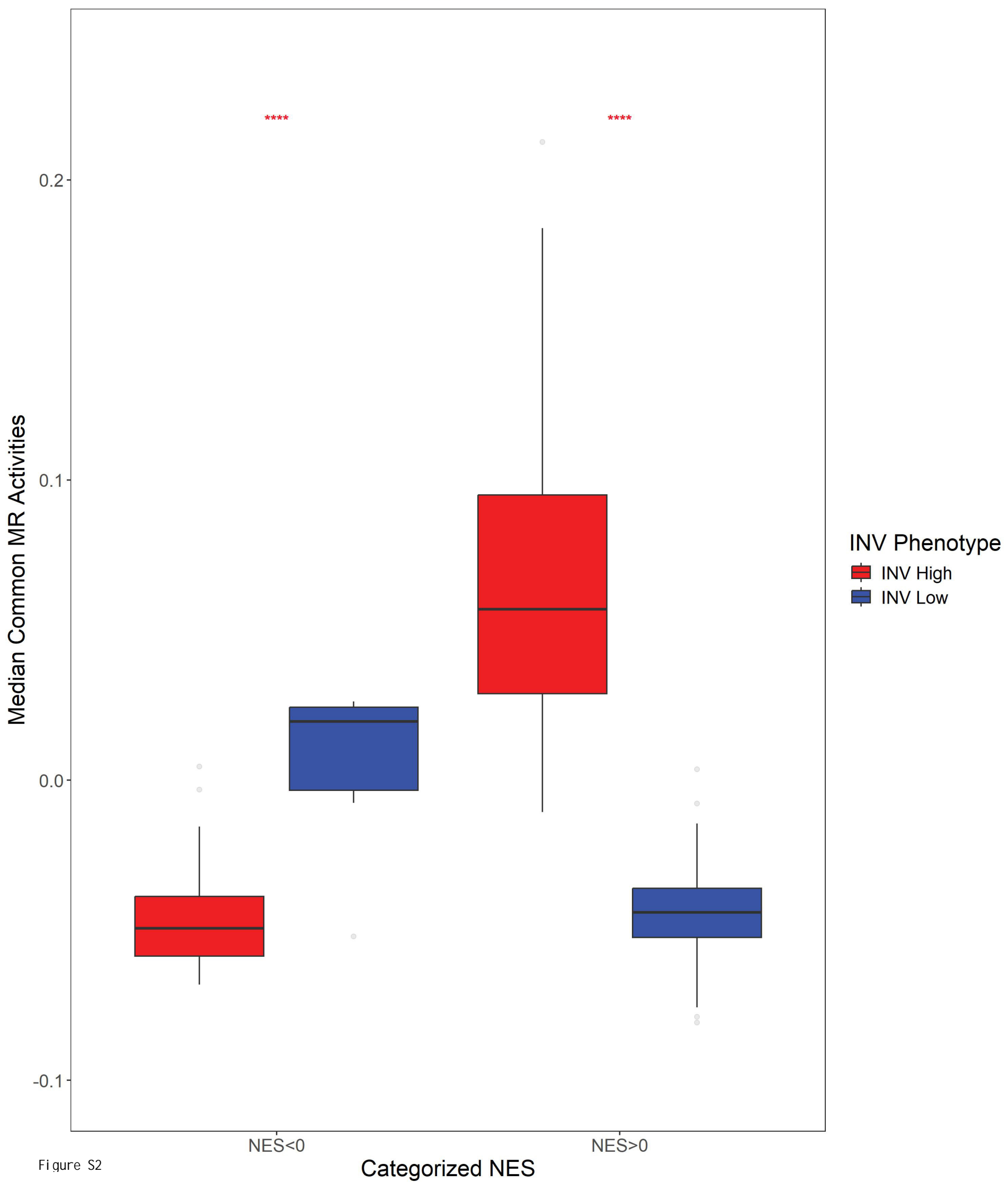

Supplement: Supplementary file 1 [file DataSheet2.pdf]

# IncNodePurity Scores for Selected Genes

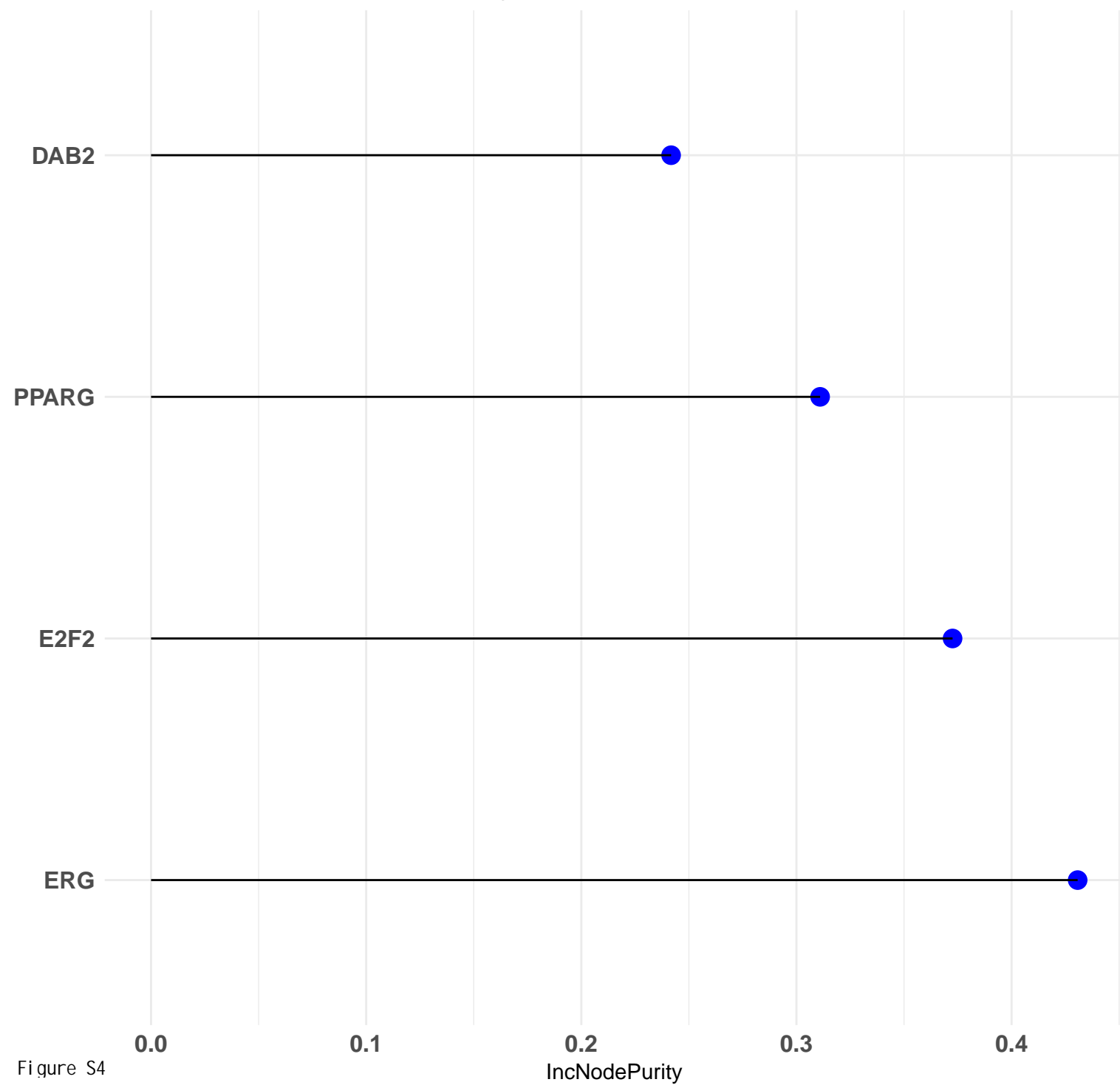

Figure S4

Supplement: Supplementary file 2 [file DataSheet4.pdf]

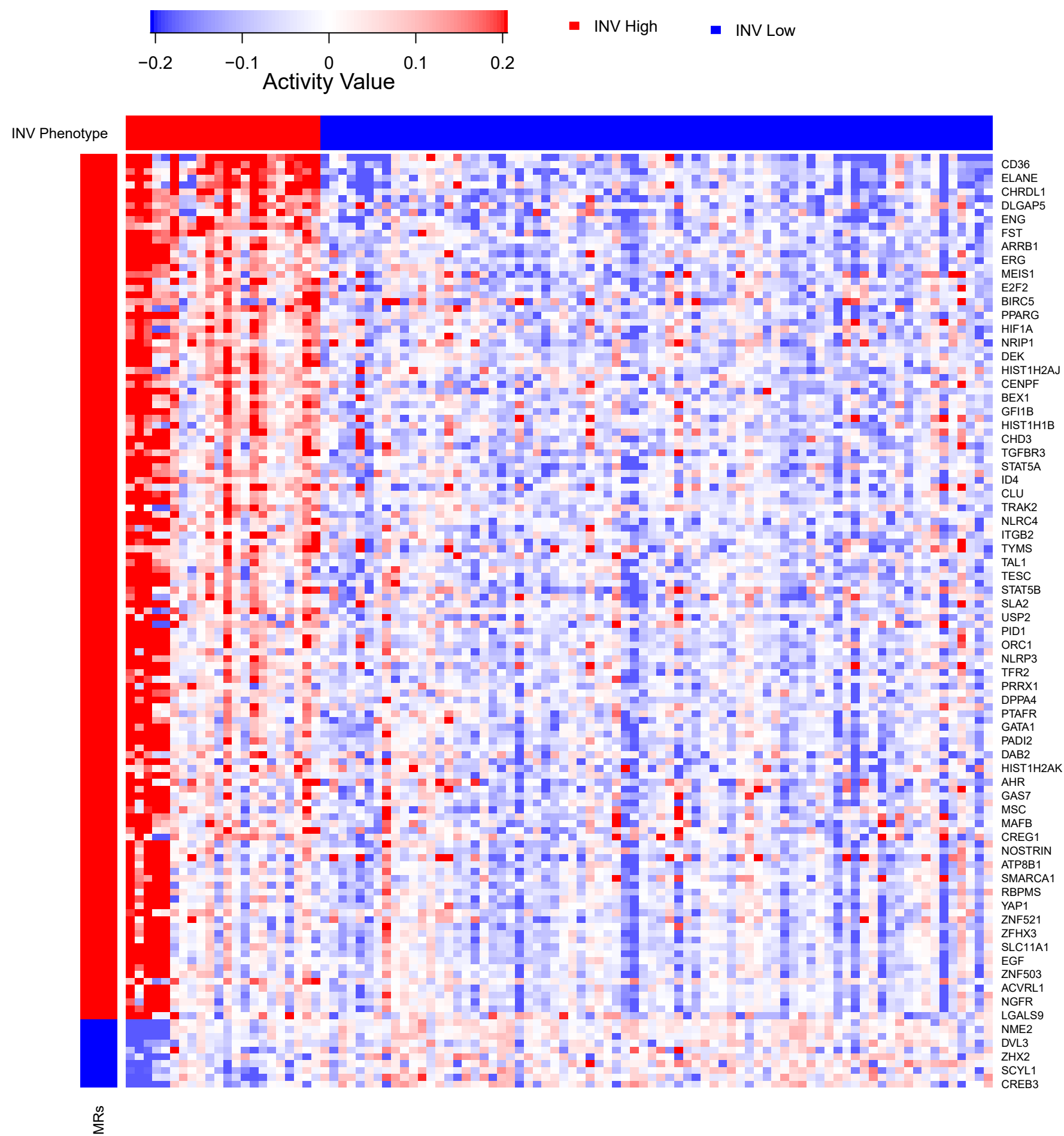

Figure S3

Supplement: Supplementary file 4 [file DataSheet3.pdf]

# Quantile-Normalized Primary Tumor Samples

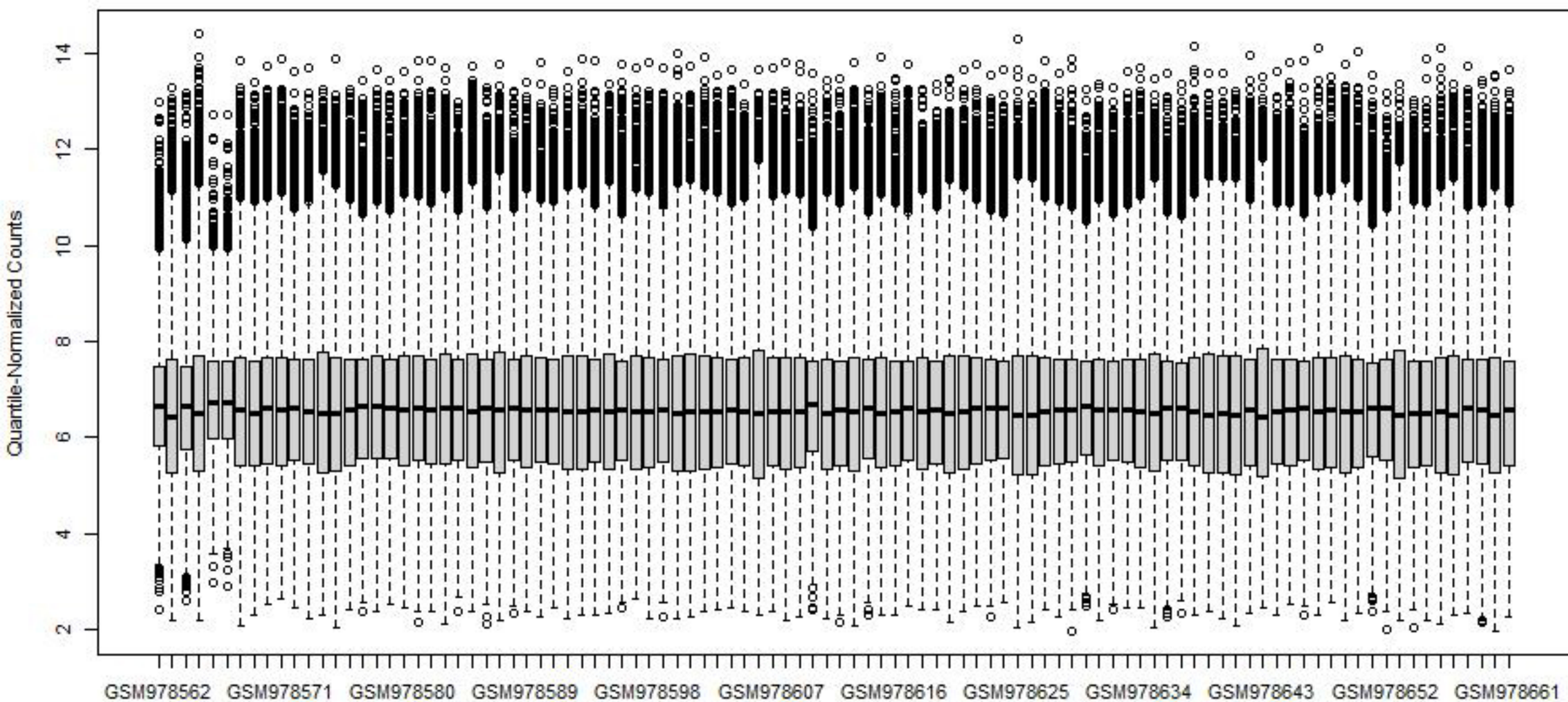

Figure S1

Supplement: Supplementary file 6 [file DataSheet1.pdf]

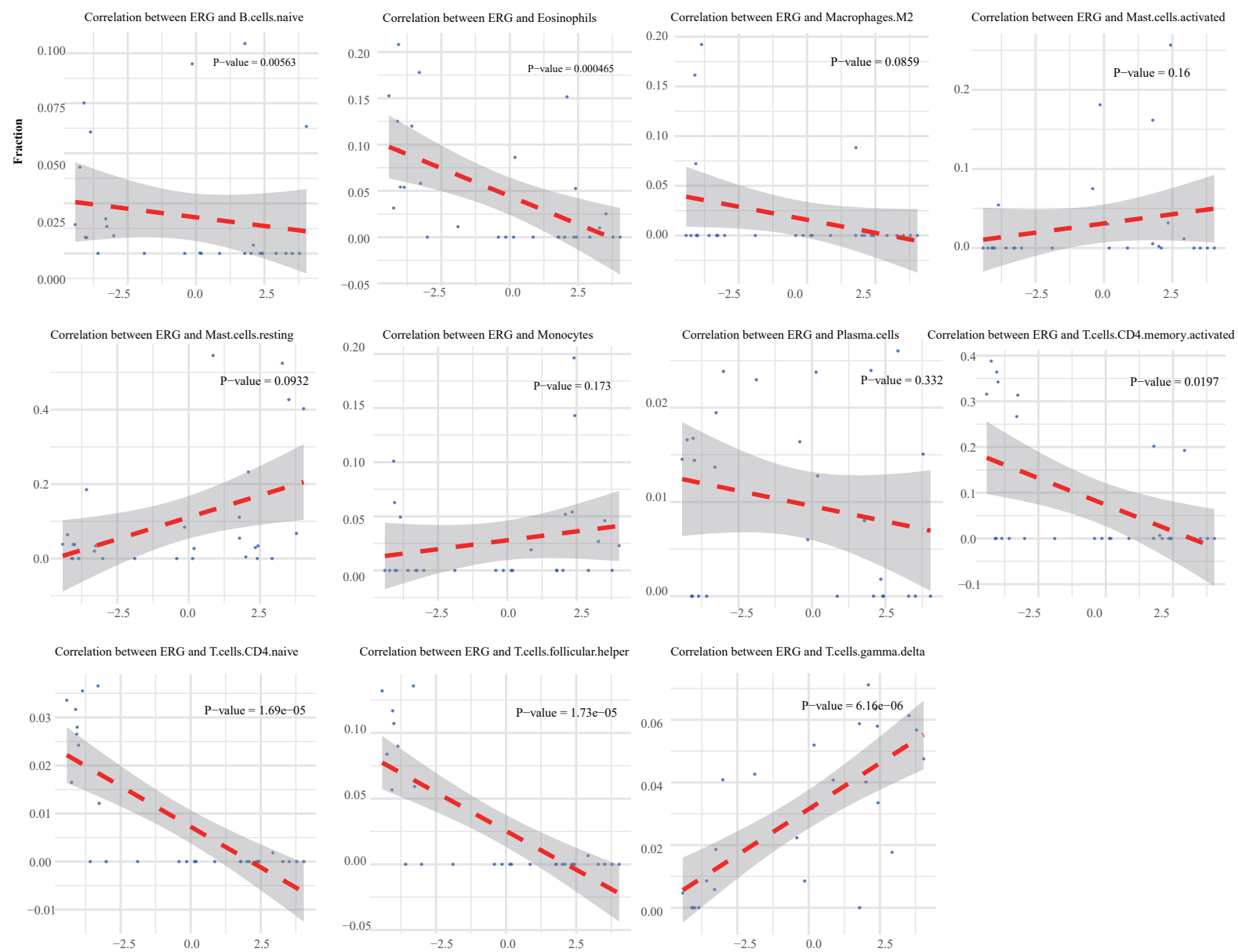

Figure S5

Supplement: Supplementary file 7 [file DataSheet5.pdf]
