# Supplementary material for: Network analysis of master regulators associated with invasive phenotypes in multiple myeloma
Source: Front Cell Dev Biol. 2025 Jul 16;13:1586870. doi: 10.3389/fcell.2025.1586870 (PMC12307296; doi:10.3389/fcell.2025.1586870)
Supplement: Supplementary file 3 [file Table1.docx]

Table 1. Candidates for drug repositioning.

| Drugs | Interacting residues of ERG | Binding affinity (kcal/mol) of ERG | Groups | Background |
| --- | --- | --- | --- | --- |
| Idarubicin | Trp142, Ala129, His202, Val208 | -9.4 | Approved | An orally administered anthracycline antineoplastic. The compound has shown activity against breast cancer, lymphomas and leukemias, together with the potential for reduced cardiac toxicity.  Targets: TOP2B, TOP2A, DNA. |
| Mitonafide | Val125, Trp145, Leu203, His191, Leu195, Ser205 | -8.1 | Investigational | Mitonafide is an antitumor agent that can be used in cancer research, such as non-small cell lung cancer and leukemia.  Targets: none. |
| Homidium bromide | Val127, Ile126, Ala129, Trp145, Leu195 | -7.9 | Investigational | This compound is a group II compound with trypanocidal effects.  Target: none. |
| Acridinamine | Arg124, Val125, Trp145, Ser205 | -7.6 | Experimental | Aminacrine is a slow-acting germicide effective against bacteria and fungi which is included in drug formulations for its anti-septic properties.  Target: none. |
| Pergolide mesylate | Arg124, Val127, Pro128, Trp145, Leu195 | -7.5 | Approved | A medication that has been used for Parkinson's Disease.  Target: DRD2, HTR1A, ADRA1B. |
