# Supplementary material for: Network analysis of master regulators associated with invasive phenotypes in multiple myeloma
Source: Front Cell Dev Biol. 2025 Jul 16;13:1586870. doi: 10.3389/fcell.2025.1586870 (PMC12307296; doi:10.3389/fcell.2025.1586870)
Supplement: Supplementary file 5 [file Table2.docx]

Table 2. The physicochemical and pharmacokinetic properties typical for three small-molecule drugs.

| Drugs | LogP | HBD | HBA | Mw | PSA | Rotatable bonds | Intestinal Absorption |
| --- | --- | --- | --- | --- | --- | --- | --- |
| Idarubicin | 1.69 | 5 | 10 | 497.5 | 176.61 Å2 | 3 | + |
| Mitonafide | 2.00 | 0 | 5 | 313.3 | 86.4 Å2 | 3 | - |
| Homidium bromide | 0.83 | 2 | 2 | 314.4 | 55.92 Å2 | 2 | - |

Note: LogP, lipid-water partition coefficient; HBD, hydrogen bond donor; HBA, hydrogen bond acceptor; MW, molecular weight; PSA: Polar Surface Area.
